# PLA2G6-Associated Neurodegeneration: A Rare Case Report of Neurodegeneration with Brain Iron Accumulation in Children

**DOI:** 10.12669/pjms.39.1.6801

**Published:** 2023

**Authors:** Nurani Widianti, Meta Herdiana Hanindita, Nur Aisiyah Widjaja, Roedi Irawan

**Affiliations:** 1 Nurani Widianti, Department of Child Health, Faculty of Medicine, Universitas Airlangga, Dr. Soetomo General Hospital, Surabaya, Indonesia; 2 Meta Herdiana Hanindita, Department of Child Health, Faculty of Medicine, Universitas Airlangga, Dr. Soetomo General Hospital, Surabaya, Indonesia; 3 Nur Aisiyah Widjaja, Department of Child Health, Faculty of Medicine, Universitas Airlangga, Dr. Soetomo General Hospital, Surabaya, Indonesia; 4 Roedi Irawan, Department of Child Health, Faculty of Medicine, Universitas Airlangga, Dr. Soetomo General Hospital, Surabaya, Indonesia

**Keywords:** Developmental regression, NBIA, PLAN, Inherited metabolic diseases

## Abstract

This report aimed to describe and review the clinical features, neuroimaging findings, and PLA2G6 mutations identified in a 34-month-old girl with regression of developmental milestones.

A 34 months old girl came to Dr.Soetomo Hospital’s outpatient clinic, Surabaya, with a developmental regression for six months, and got worse until she could not do any activity and was followed by recurrent seizures. She had a sibling who had similar problems and symptoms and then died at five years of age. The head MRI revealed brain atrophy, the possibility of an early sign of metabolic disorder, and a white matter lesion at the globuspallidus bilateral that supports the encephalopathy metabolic view. The genetic test revealed a positive homozygous such as a pathogenic variant in the PLA2G6 gene, which confirmed the diagnosis. PLA2G6-Associated with Neurodegeneration (PLAN) should be considered as a diagnosis in children with developmental regression.

## INTRODUCTION

Over the past two decades, nearly all Neurodegeneration with Brain Iron Accumulation (NBIA) disorders have been elucidated, particularly with a focus on the basal ganglia and globus pallidus.^1^ NBIA is a set of life-threatening rare monogenic diseases characterized by focal iron accumulation in the brain, with a prevalence of less than 1/1,000,000 in the general population and considered a “rare” disease.^2^ NBIA is divided into four types: pantothenate kinase-associated neurodegeneration (PKAN); phospholipase A2-associated neurodegeneration (PLAN); mitochondrial membrane protein-associated neurodegeneration (MPAN); and beta-propeller protein-associated neurodegeneration (BPAN). Each type gives a different presentation and prognosis.^2^

The present paper reports a case of NBIA PLAN type in a 34-month-old girl. To the best of our knowledge, this is the first reported case of neurodegeneration with brain iron accumulation in Indonesia. The purpose of this case report was to report and review the disease. This report emphasizes the importance of correct diagnosis for a rare case of neurodegeneration with brain iron accumulation in a child, thus we can monitor the patient better, reduce the mortality rate, improve the patient’s quality of life and estimate their prognosis better.

## CASE PRESENTATION

A 34-month-old girl came to Dr.Soetomo Hospital’s outpatient clinic, Surabaya, on 14 June 2021, with a chief complaint of developmental regression since she was eighteen months old. The problems worsened until she could not do any activity when she was 24 months old. She experienced recurring seizures without fever twice a month.

Her four-years-older-sibling died at the age of five after showing similar signs and symptoms. The patient was delivered by C-section at 38 weeks of gestational age, and the indication of breech presentation with birth weight, length, and head circumference were 2900 grams, 47 cm, and 32 cm, respectively. The patient had completed the basic immunization. According to her nutritional history, the mother breastfed her until she was 18 months old, continued with the formula milk via spoon until 32 months old, and then enteral from 33 months old until now.

She could smile at two months old, prone at four months old, babble at six months old, say “mama” specifically, and clap hands at nine months old. At nine months old, she could not sit down, head up, prone herself, or reach toys. At 18 months old, her babbling was decrement, and she fell while sitting, prone rarely but still could head up and reach a toy. At 24 months old, she could not head up, inability to be prone by herself, could not reach toys, there was no babbling but still could cry, and her eyes still could follow a motion. The upper extremities could move flexion, and the lower extremities could move laterally. Furthermore, day by day, she could only cry softly.

The vital sign and physical examination showed no remarkable findings. A BCG scar was found on the right arm. Her neurological status revealed apathy, isochoric pupils of 3mm/3mm, positive light reflex, and no sign of meningeal irritation. Physiological reflex decreased +1 for all extremities and no pathological reflex. The motor examination resulted in +1 for all extremities and flaccid muscle tone.

The laboratory finding revealed that Hb 11.6 g/dl, Hct 35.8 g/dl, MCV 71.6 fL, MCH 23.2 pg, MCHC 32.4 g/dL, leucocyte 9290 uL, thrombocyte 532000 uL, erythrocyte 5000000 uL, Ca 9.6 mg/dL, sodium 137.2 mmol/L, potassium 3.81 mmol/L, chloride 105.7 mmol/L, pH 7.42, pCO2 23.4 mmHg, pO2 79 mmHg, SO2 96%, BE -9 mmol/L, T 37.5C, and lactic acid 4.7 mmol/L, and ammonia 62.2 mmol/L.

**Fig.1:**
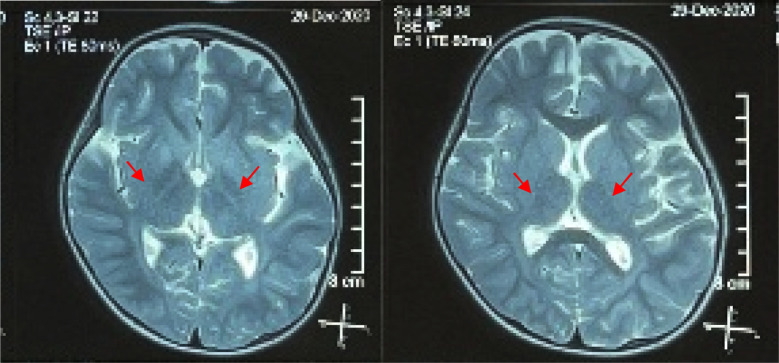
The second head MRI showed a white matter lesion at globus pallidus bilateral that supports the encephalopathy metabolic view (new lesion), subdural fluid collection on bilateral frontal regions, focal brain atrophy fronto-temporo-parietal bilateral lobe, cerebellum, and brain stem (increasing process), and microcephalic with brachycephalic skull type.

The amino profile-19 examination showed a decrement in aspartic acid (ASP) and an increment in glutamic acid (GLU). The first head MRI on December 2019 revealed slight brain atrophy, which had the possibility of an early sign of a metabolic disorder of the brain. At this time, there are no pathognomonic signs of metabolic disease in the brain parenchymal yet. The second head MRI was performed one year later and revealed a white matter lesion at globus pallidus bilateral that supports the encephalopathy metabolic view (new lesion), subdural fluid collection on bilateral frontal regions, focal brain atrophy fronto-temporo-parietal bilateral lobe, cerebellum, and brain stem (increasing process), and microcephalic with brachycephalic skull type. (Fig.1).

The electroencephalography on December 2nd 2020 showed abnormal EEG III, spike-wave in the left mid-temporal, and excessive beta activity. The impression of abnormal EEG during recording indicated an epileptogenic potential in the left mid-temporal region.

A genetic test was performed on April 1st 2021. The interpretation was that a homozygous possible pathogenic variant was identified in the PLA2G6 gene. She was diagnosed with Neurodegeneration with Brain Iron Accumulation (NBIA): PLA2G6-Associated Neurodegeneration (PLAN) type. Symptomatic treatment was administered to the patient. Informed consent for the publication of clinical data has been obtained from this patient.

## DISCUSSION

**Fig.2:**
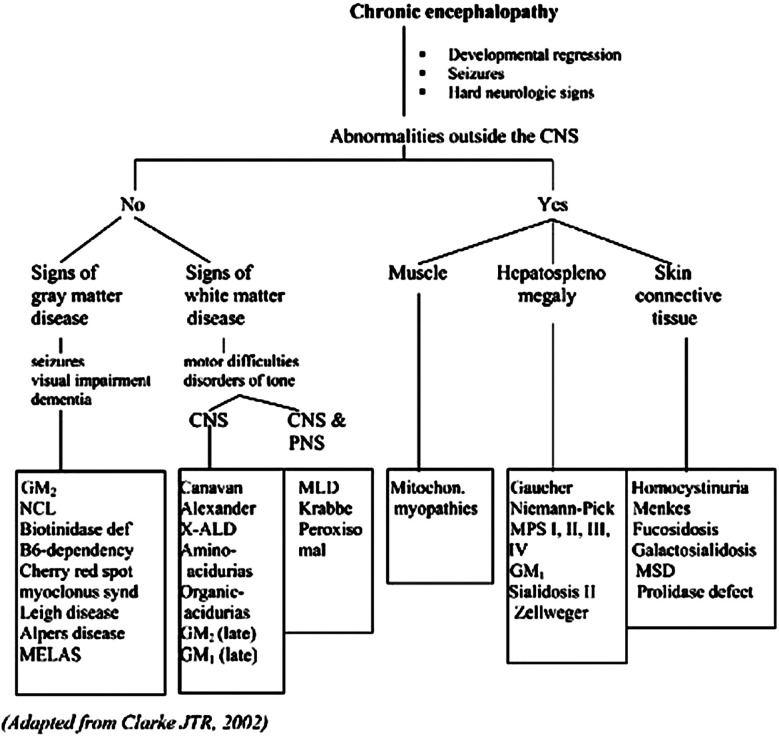
Approach to inherited metabolic diseases with chronic encephalopathy.^3^ ***Abbreviations:*** **GM2:** gangliosidosis, **GM1:** gangliosidosis, **NCL:** neuronal ceroidlipofuscinosis, **MELAS:** mitochondrial encephalopathy lactic acidosis syndrome, **X-ALD:** X-adrenoleukodystrophy, **MLD:** metachromatic leukodystrophy, **MPS:** mucopolysaccharidosis, **MSD:** multiple sulfatase deficiency

In this case, a 34 months old girl came with a chief complaint of developmental regression. Careful and comprehensive clinical assessment, imaging studies, electrophysiologic investigation, and histopathologic and ultrastructural information from selected biopsies help establish the distribution and type of abnormalities within the nerve system. Retinitis pigmentosa, hepatocellular dysfunction, and renal tubular dysfunction in a child with psychomotor retardation, muscle weakness, and seizures strongly suggest the possibility of a mitochondrial defect. While hepatosplenomegaly without significant hepatocellular dysfunction in a child with slowly progressive psychomotor retardation and ataxia without seizure may suggest lysosomal storage diseases.^3^ Neurologic symptoms such as global cognitive disabilities, developmental delay, loss of previously acquired skills, or psychomotor retardation are the presenting and most major clinical problems associated with many inherited metabolic disorders.^3^(Fig.2)

Our patient had a sibling who died at five years old with similar signs and symptoms. Most mutations cause diseases transmitted as autosomal recessive disorders.^3^ Seven of the ten genetically defined types of NBIA are inherited autosomal recessive, except neuroferritinopathy, mitochondrial membrane protein-associated neurodegeneration (MPAN), and beta-propeller protein-associated neurodegeneration (BPAN).^4^ NBIA diagnosis is based on a genetic test. A biochemical test is performed to rule out a differential diagnosis, while an MRI is performed as an additional examination to confirm the diagnosis.

Patients with a movement disorder, extrapyramidal features, or developmental regression were recommended, including iron-sensitive sequences in diagnostic MRI studies of the brain, although iron, resembling the eye of the tiger sign, might be visible only later in the disease course.^4^ The appearance of specific MRI patterns depends on the stage of the disease and the patient’s age at evaluation.^5^ In all PLAN forms, iron accumulation might be absent or subtle early in the disease course.

Optic atrophy can be seen in up to 85% of patients with PLA2G6 mutation.^2^ A case series from India present a girl with rotatory and horizontal nystagmus with optic disc atrophy in both eyes and a boy with nystagmus with bilateral disc pallor.^6^ Another study in 2021 presented a girl with bilateral optic nerve atrophy (without retinal hyperpigmentation).^7^ An abnormal iron deposition follows a specific pattern of almost symmetrical involvement of the deep grey matter nuclei of the brain in NBIA.^8^ Neurodegeneration with brain iron accumulation (NBIA) encompasses a group of inherited disorders that share the clinical features of an extrapyramidal movement disorder accompanied by varying degrees of intellectual disability and abnormal iron deposition in the basal ganglia (e.g., globuspallidus mainly).^2^ A case study from Hungary showed the development of severe spastic paraparesis with moderate ataxia in two girls diagnosed with PLAN.^7^

Mutations in the calcium-independent phospholipase A2 gene *PLA2G6*, which plays a critical role in cell membrane phospholipid homeostasis, are responsible for PLAN. To date, all defective proteins in the NBIA disorders are known or suspected to be essential for mitochondrial function, but only two have a known role in iron homeostasis: ferritin light chain and ceruloplasmin.^1^

As whole-exome sequencing emerged, many cases of PLAN have been recognized.^1^ Iron is an essential element for many brain functions^9^, particularly for myelination. However, high amounts of iron cause neural damage due to the ability of labile forms of iron to induce oxidative stress and provide a variety of neurodegenerative diseases.^10^ NBIA involves several genetic disorders caused by mutations in genes directly involved in an iron metabolic pathway.^9^ The four most common NBIA disorders include PKAN due to mutations in PANK2, phospholipase A2-associated neurodegeneration caused by a mutation in PLA2G6, mitochondrial membrane protein-associated neurodegeneration from mutations in C19orf12, and beta-propeller protein-associated neurodegeneration due to mutations in WDR45.^1^

Childhood-onset PLAN encompasses a more slowly progressive psychomotor disorder that is clinically distinct from the infantile and adult-onset PLAN forms. These children may initially be diagnosed with autism, eventually developing motor features; most have some degree of intellectual disability.^1^ Movement disorders may be alleviated using anticholinergics, tetrabenazine, and baclofen dopaminergic drugs. Surgical procedures, thalamotomy, pallidotomy, or deep brain stimulation might be beneficial in about 30% (for review of reported cases).^11^ When the individual could no longer maintain an adequate diet orally, gastrostomy tube placement is indicated to prevent aspiration pneumonia.^4^

The possibility of chelating iron accumulated in specific brain regions remains an open question,^10^ but there are three reports on chelating treatment in idiopathic NBIA patients with clinical benefit, thus unknown diagnosis may not necessarily prevent initiation of chelating treatment.^10^,^12^ It is appropriate to offer genetic counselling (including discussion of potential risks to offspring and reproductive options) to young adults who are affected, are heterozygous, or are at risk of being heterozygous. When the NBIA-causing pathogenic variant(s) have been identified in an affected family member, prenatal testing for a pregnancy at increased risk is highly recommended.^4^

NBIA is a very rare case and it is very challenging to make the diagnosis. However, it is important to make a diagnosis as early as possible so that patients can get treated and monitored better (especially in ferritin levels as an iron deposition). This condition will also reduce the mortality rate, improve quality of life, and help us to estimate their prognosis better.
